# C1QTNF6 is a Prognostic Biomarker and Related to Immune Infiltration and Drug Sensitivity: A Pan-Cancer Analysis

**DOI:** 10.3389/fphar.2022.855485

**Published:** 2022-03-23

**Authors:** Wei Liu, Jian Zhang, Tao Xie, Xiaoting Huang, Baiyao Wang, Yunhong Tian, Yawei Yuan

**Affiliations:** Department of Radiation Oncology, Affiliated Cancer Hospital and Institute of Guangzhou Medical University, Guangzhou, China

**Keywords:** pan-cancer analysis, C1QTNF6, prognosis, immune infiltration, tumor microenvironment, drug sensitivity

## Abstract

**Background:** The discovery of reliable cancer biomarkers could tune a diagnosis and improve the way patients are treated. However, many cancers lack robust biomarkers. *C1QTNF6* has been preliminarily elucidated for its role in some tumors. However, no pan-cancer analysis has been performed to comprehensively explore the value of *C1QTNF6*.

**Methods:** Data from the TCGA database, GTEx database stored in the USUC Xena were used for analyzing the profiles of *C1QTNF6* expression in normal and tumor tissues in pan-cancer. Subsequently, the gene alteration rates of *C1QTNF6* were acquired on the online web cBioportal. With the aid of the TCGA data, the association between *C1QTNF6* mRNA expression and copy number alterations (CNA) and methylation was determined. Survival analyses of *C1QTNF6* were carried out. Moreover, the tumor biological and immunological characteristics of *C1QTNF6* were clarified in the forms of the correlation between *C1QTNF6* expression and hallmark Pathway scores in MsigDB database, immune cell infiltration, immune-related genes. We conducted a GSEA of *C1QTNF6* to illustrate its potential biological functions. In addition, GDSC2 data with 198 drugs were adopted to explore drug sensitivity with the change of *C1QTNF6* expression.

**Result:** *C1QTNF6* was overexpressed in many types of cancer, Survival analysis showed that *C1QTNF6* independently served as a prognostic indicator for poor survival in many tumors. Besides, we also identified a positive correlation between *C1QTNF6* and cancer hallmark pathway score, tumor microenvironment related pathways score (TMEp score), and immune characteristic. In terms of drug sensitivity analysis, we found higher expression level of *C1QTNF6* predicts a high IC50 value for most of 198 drugs which predicts drug resistance.

**Conclusions:** Our study provides a new biological marker for pan-cancer, which is beneficial to the diagnosis and treatment of cancer, which bring a new therapeutic target for tumors.

## Introduction

At present, cancer remains a hindrance in the field of medicine (Hausman, 2019). Compared to normal cells, cancer cells have several unique hallmarks, including unlimited replication, overactivation of growth signals, resistance to cell death, immune re-editing, and metabolic reprogramming (Hanahan and Weinberg, 2011; Macheret and Halazonetis, 2015). These changes are the result of mutations in a variety of genes, including proto-oncogenes and suppressor genes (Kroemer and Pouyssegur, 2008; Park et al., 2020). We hypothesized that these genes may serve as viable therapeutic targets in cancer treatment. Targeted therapies such as BRAF inhibitors and VEGF inhibitors have achieved initial efficacy in the treatment of diverse solid tumors (Davies et al., 2002; Carmeliet, 2005). Besides, immunotherapy based on *PD-1/PD-L1* or *CTLA-4* mutations offers hope for advanced tumors (Ai et al., 2020; Han et al., 2020). However, unfortunately, a significant proportion of patients are less sensitive to targeted therapy or immunotherapy (Qin et al., 2019). Therefore, there is an urgent need for new biomarkers to guide cancer treatment.

Complement C1q Tumor Necrosis factor-related Protein 6 (*C1QTNF6*) is an inflammation-related gene, which has been preliminarily described to be related to the biological characteristics of tumors (Wang et al., 2020). Zhang et al. found that *C1QTNF6* regulates proliferation and apoptosis of non-small cell lung cancer (NSCLC) (Zhang and Feng, 2021). Takeuchi et al. found that *C1QTNF6* regulates angiogenesis and apoptosis in hepatocellular carcinoma through the Akt pathway (Takeuchi et al., 2011). Therefore, *C1QTNF6* might perform an integral function in tumor progression. Nevertheless, no pan-cancer analysis has been performed to comprehensively demonstrate the value of *C1QTNF6* in multiple cancers.

In recent years, the establishment of several large public databases has greatly promoted the development of bioinformatics. TCGA and GTEx databases are among the most used databases (Blum et al., 2018). By analyzing cancer transcriptome data and clinical data, we can screen for meaningful biomarkers to assess patient prognosis or guide treatment.

Here, bioinformatics analysis was conducted for the purpose of comprehensively examining the significance of *C1QTNF6* in pan-cancer by combining GTEx and TCGA data. Our results can provide a robust prognostic marker for multiple cancers. The expression level of *C1QTNF6*, as well as its prognostic significance in pan-cancer, was explored. We further found the role of *C1QTNF6* in the tumor microenvironment (TME), including the correlation between *C1QTNF6* and the immunosuppressive genes, immune cell infiltration score, chemokines, and chemokine receptors. It is worth noting that increased *C1QTNF6* expression indicates greater drug resistance. Taken together, our research can also provide a reference for the understanding of the cancer-immune microenvironment and cancer therapies.

## Materials and Methods

### Data Download and Expression Analysis

RNA-sequencing samples of the Genotype-Tissue Expression (GTEx) and The Cancer Genome Atlas (TCGA) were acquired through UCSC Xena in TCGA TARGET GTEx columns (https://xena.ucsc.edu). We downloaded the methylation data and DNA copy number data from cBioportal dataset (https://www/cbioportal/org/). The differences in the expression levels between tumors and normal tissues were assessed by means of the T-test, and a *p*-value of less than 0.05 denoted a significance. The analysis was conducted with the help of R software.

### Gene Mutation Analysis and Methylation Analysis

First, mutation analysis was performed on the cBioportal online web. On the website, the gene was selected as *C1QTNF6*, and the object of analysis was set as “TCGAPan-Cancer Atlas”. The frequency analysis of *C1QTNF6* alteration in each cancer was obtained. The cBioPortal database was utilized for the purpose of acquiring Copy number alterations (CNA) and methylation data, while methylation data are from the HM450 types, both the association between the *C1QTNF6* expression and the CNA and methylation were calculated the Pearson’s correlation coefficients.

### Survival Analysis

Next, a univariate Cox regression was performed to explore the prognostic significance of *C1QTNF6* in pan-cancer. We also analyzed the progression-free interval (PFI), disease-free interval (DFI), disease-specific survival (DSS), and overall survival (OS). Meanwhile, univariate cox regression and multivariate Cox regression analysis of *C1QTNF6* and other cliniacal features were carried out in tumors where *C1QTNF6* was an independent prognostic indicator. The R package “Survminer” was used for analysis (Biecek, 2020).

### Analysis of C1QTNF6 in Tumor Microenvironment

The MsigDB database contains 50 hallmark Pathway datasets. First, Combining the RNA-seq data of 33 cancers and a single-sample gene set enrichment analysis (ssGSEA) algorithm utilizing GSVA packages (Hänzelmann et al., 2013), we quantified the score of each cancer hallmark. The immune characteristic parameters including ImmuneScore, StromalScore, and ESTIMATEScore of each cancer were calculated by the “ESTIMATE” R package (Verhaak, 2016). We adopted the algorithm that is published in Nature journal and “prcomp” packages to calculate the tumor microenvironment pathways score (TMEp score). The immueCellAI database was utilized for the purpose of acquiring the scores of infiltrating immune cells from the TCGA pan-cancer (http://bioinfo.life.hust.edu.cn). A total of 33 cancers were studied for their gene expression levels of chemokines, chemokine receptors, and immunosuppressive genes. After obtaining the above scores as well as the immune-associated genes expression level (chemokines, chemokine receptors, and immunosuppressive genes) of each tumor, we used the “corrplot” R package (Simko, 2021) to calculate the correlation between *C1QTNF6* and above scores and immune-related genes expression, and used the “ggplot2” package to draw the correlation heat map (Wickham, 2016).

### Gene Set Enrichment Analysis of C1QTNF6 in Pan-Cancer

GSEA was launched to explore the potential biological functions of *C1QTNF6* in the tumor process by using “clusterProfiler” (Yu et al., 2012), The top 20 significant pathways of *C1QTNF6* were obtained in each cancer, we showed GSEA plots of the 8 cancers with the largest proportion of immune pathways with the help of “ggridges” packages (Wilke, 2021).

### Drug Sensitivity Analysis

We explored the relationship between gene expression and drug sensitivity from the GDSC2 database (https://www.cancerrxgene.org/). Subsequently, Spearman- correlation analysis was employed for the purpose of investigating the correlation between drugs and *C1QTNF6* expression level, and the drugs with the top 6 strongest positive correlation and the top 6 strongest (only 6) negative correlation were displayed respectively. Next, we analyzed the difference in sensitivity between the low- and high-expression group (according to the median value of *C1QTNF6*) to 9 commonly used anticancer drugs, we assessed the sensitivity difference by the Kruskal-Wallis rank-sum test (*p* < 0.05).

## Results

### The Expression of *C1QTNF6* in Pan-Cancer

**FIGURE 1:**
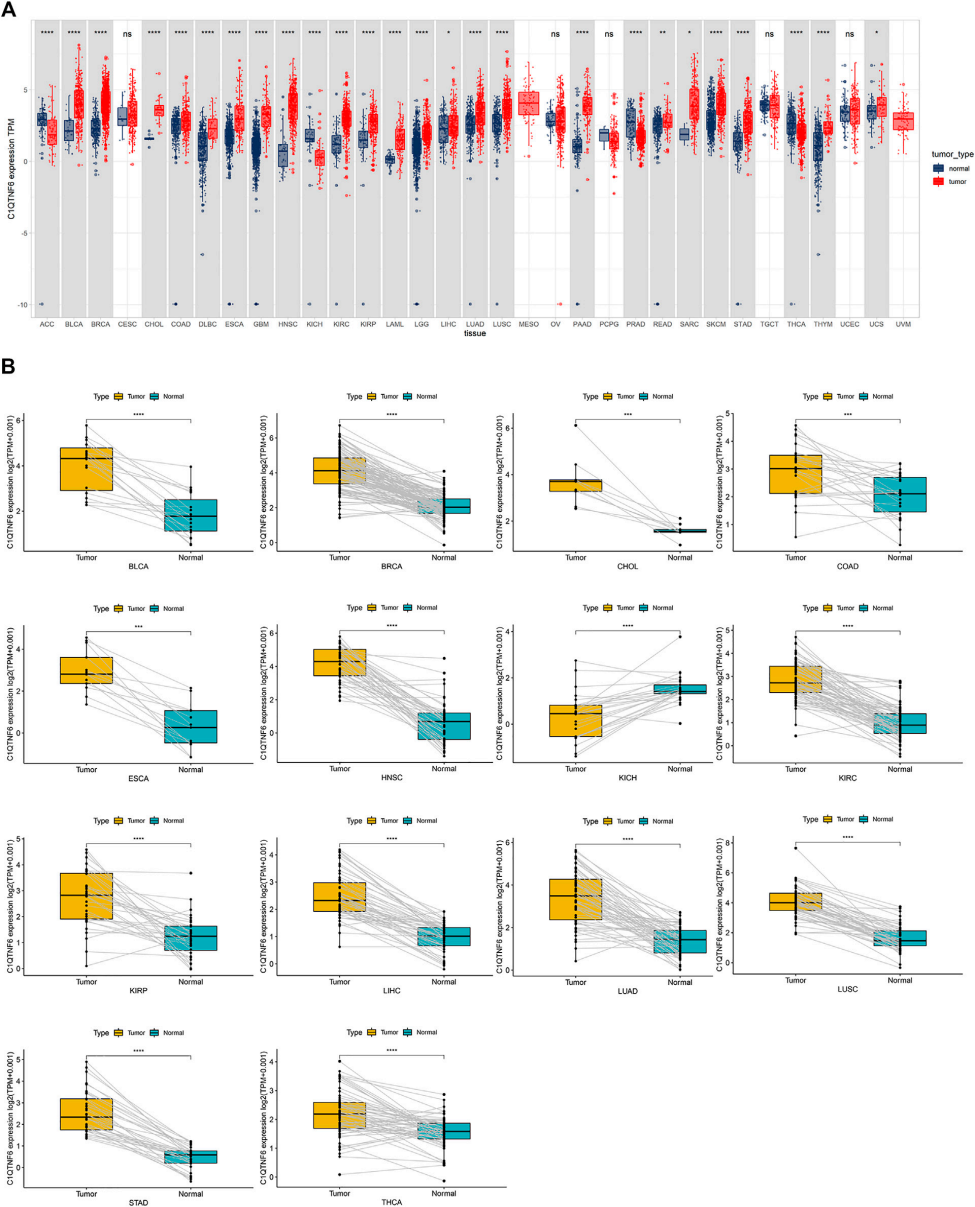
C1QTNF6 profile in pan-cancer. **(A)** Integrated GTEx database and TCGA database revelation of C1QTNF6 expression across normal and tumor tissues. **(B)** According to the TCGA data, the levels of C1QTNF6 in corresponding tumors and normal tissues varied across various cancers. *p < 0.05, **p < 0.01, ***p < 0.001, ****p < 0.0001.

*C1QTNF6* was differentially expressed in 26 cancers in the TCGA cohort. The *C1QTNF6* expression level was remarkably up-regulated in 22 distinct types of tumors, including UCS, CHOL, STAD, DLBC, SARC, GBM, PAAD, HNSC, LUSC, KIRC, LUAD, KIRP, LIHC, LGG, LAML, ESCA, READ, COAD, SKCM, BRCA, THYM, BLCA. *C1QTNF6* was significantly down-regulated in 4 tumors, including ACC, KICH, PRAD, and THCA; The cancers in the gray box had significant differences between tumor and normal tissues, whereas cancers in the white box had no differences (Figure 1A) Subsequently, paired expression analysis showed *C1QTNF6* was significantly up-regulated in the 13 kinds of tumors, notably, KIRP, HNSC, LIHC, ESCA, LUAD, COAD, KIRC, LUSC, CHOL, STAD, BRCA, THCA, BLCA. Paired expression analysis showed that *C1QTNF6* was solely significantly down-regulated in KICH (Figure 1B).

### Gene Mutation Analysis and Methylation Analysis

**FIGURE 2:**
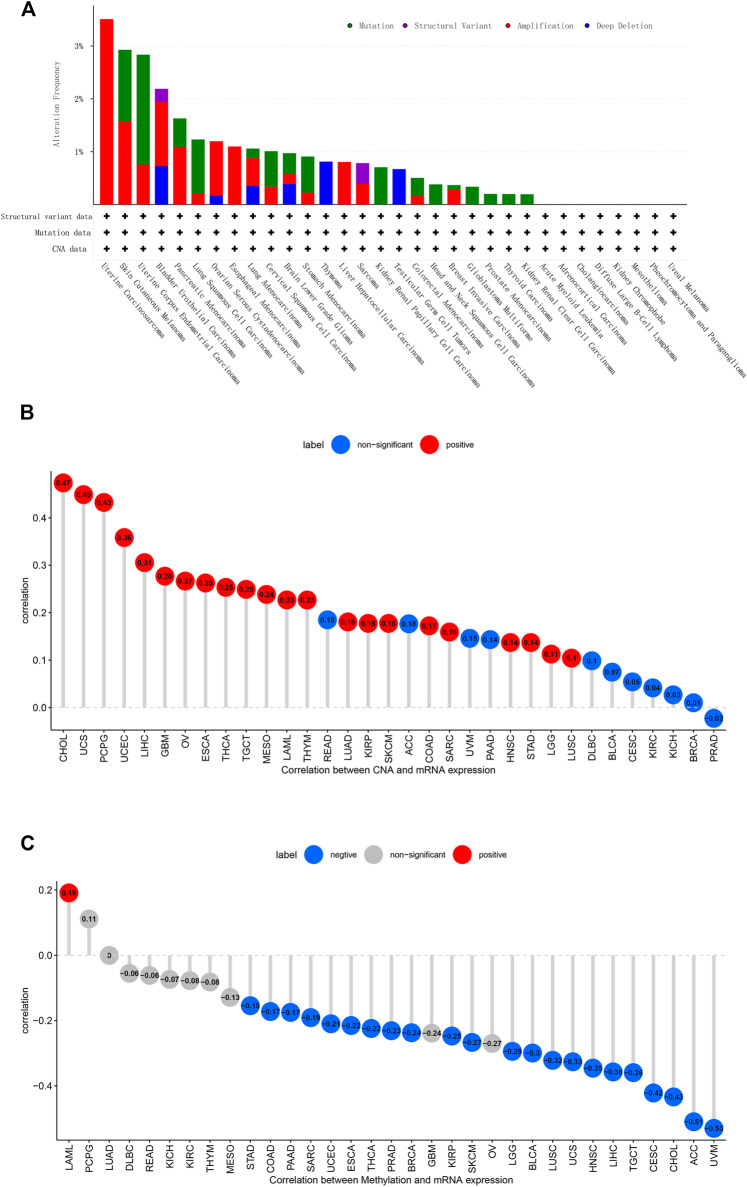
CNA and DNA methylation of C1QTNF6 in pan-cancer. **(A)** alteration frequency of C1QTNF6 in each cancer of TCGA. **(B)** C1QTNF6 expression and DNA copy number correlation profile in each cancer of TCGA. The deeper red color means significant cancer types (*p* < 0.05). **(C)** correlations between C1QTNF6 expression and DNA methylation, the blue represented negatively correlated, and the red represented positively correlated.

First, analysis in the cBioportal database showed the alteration frequency of *C1QTNF6* in Pan-cancer. The results showed that the alteration frequency of *C1QTNF6* in uterine carcinosarcoma was the highest, and the main type is the amplification mutation (Figure 2A). Subsequently, we explored the association between *C1QTNF6* and copy number mutation (CNA) in pan-cancer. The results showed that *C1QTNF6* had a strong positive correlation with CNA in CHOL, UCS, PCPG (Figure 2B). Then, we explored the correlation between *C1QTNF6* and methylation in pan-cancer. The findings demonstrated that *C1QTNF6* exhibited a positive correlation with methylation in LAML, and a strong negative correlation with ACC and UVM, etc. (Figure 2C).

### Survival Analysis

**FIGURE 3:**
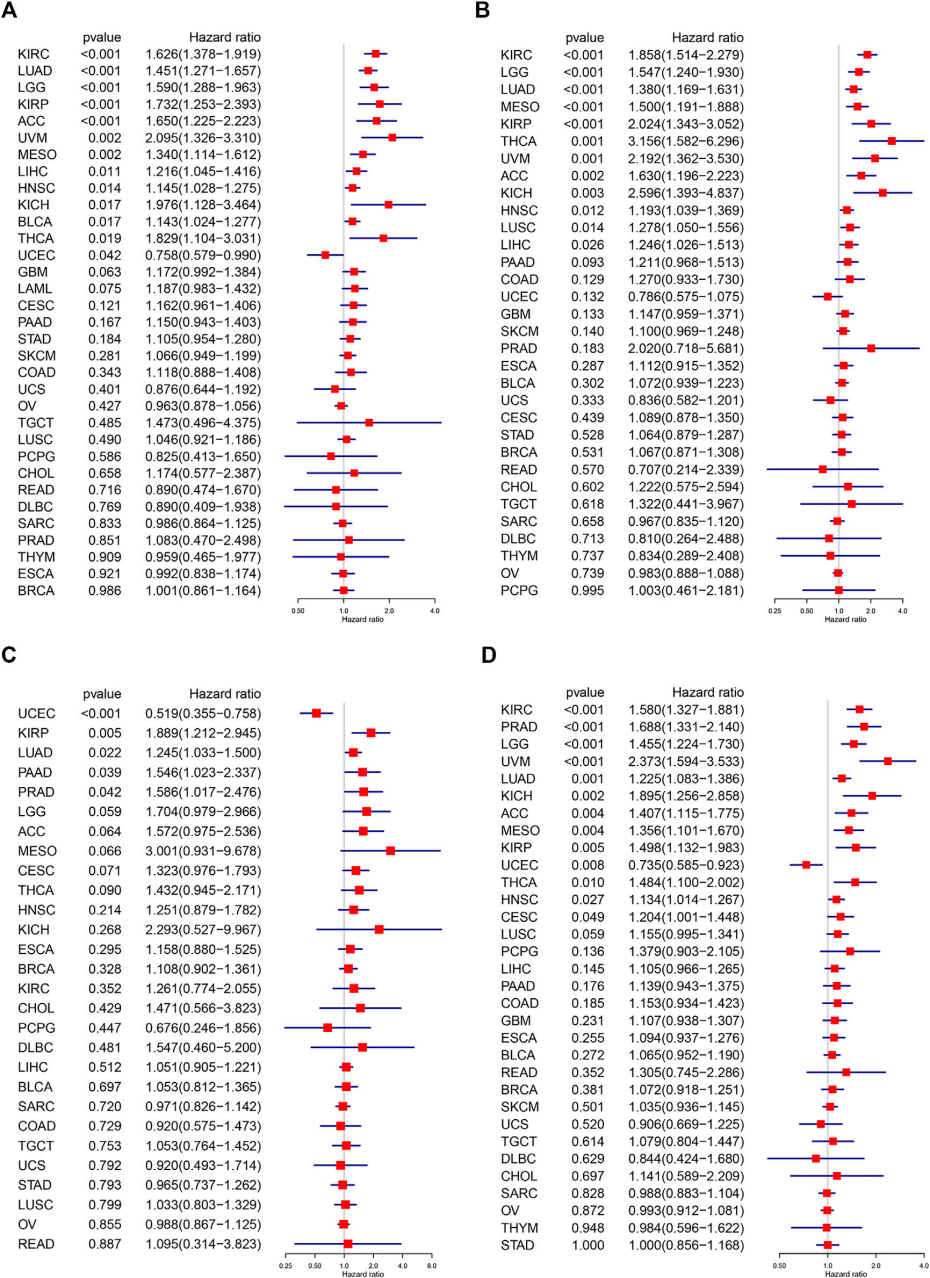
Univariate Cox regression analysis of C1QTNF6. The results were shown with a forest map for **(A)** OS; **(B)** DSS; **(C)** DFI; **(D)** PFI.

Subsequently, we investigated the prognostic significance of *C1QTNF6* in pan-cancer. We also evaluated the overall survival (OS, Figure 3A), disease-specific survival (DSS, Figure 3B), disease-free interval (DFI, Figure 3C), and Progression-free interval (PFI, Figure 3D), respectively. Univariate Cox regression was used to eliminate the confounding bias. First, in overall survival, *C1QTNF6* was an independent prognostic gene in KIRC, LUAD, LGG, KIRP, ACC, UVM, MESO, LIHC, HNSC, KICH, BLCA, THCA, and UCEC. Then, in the analysis of disease-specific survival, *C1QTNF6* was an independent prognostic gene in KIRC, LGG, LUAD, MESO, KIRP, THCA, UVM, ACC, KICH, HNSC, LUSC, and LIHC. Subsequent analysis of disease-free survival showed that *C1QTNF6* was an independent prognostic gene in UCEC, KIRP, LUAD, PAAD, and PRAD. Finally, PFS analysis showed that *C1QTNF6* was an independent prognostic gene in KIRC, PRAD, LGG, UVM, LUAD, KICH, ACC, MESO, KIRP, UCEC, THCA, HNSC, and CESC. A value of Hazard ratio greater than 1 indicates a risk prognostic factor, it is found that *C1QTNF6* predicts poor survival in most cancers. At the same time, univariate cox regression and multivariate cox regression analysis of *C1QTNF6* and clinical features were carried out in tumors which suggest *C1QTNF6* is independent prognostic factor in OS analysis, the multicox result demonstrate that *C1QTNF6* is an independent factors associated with patients overall survival time in KIRC, LUAD, LGG, KIRP, ACC, UVM, MESO, LIHC, HNSC, which is generally consistent with the unicox results (Supplementary Figures S1A–M). For instance, Univariate Cox regression analysis showed that age,stage, *C1QTNF6 were significantely associated with the prognosis of ACC,* Multivariate Cox regression analysis showed that *C1QTNF6* was an independent factor after adjusted for other clinical characteristic in ACC patients.

### Correlation Between *C1QTNF6* and Cancer Pathway (Hallmark Pathway Sets)

**FIGURE 4:**
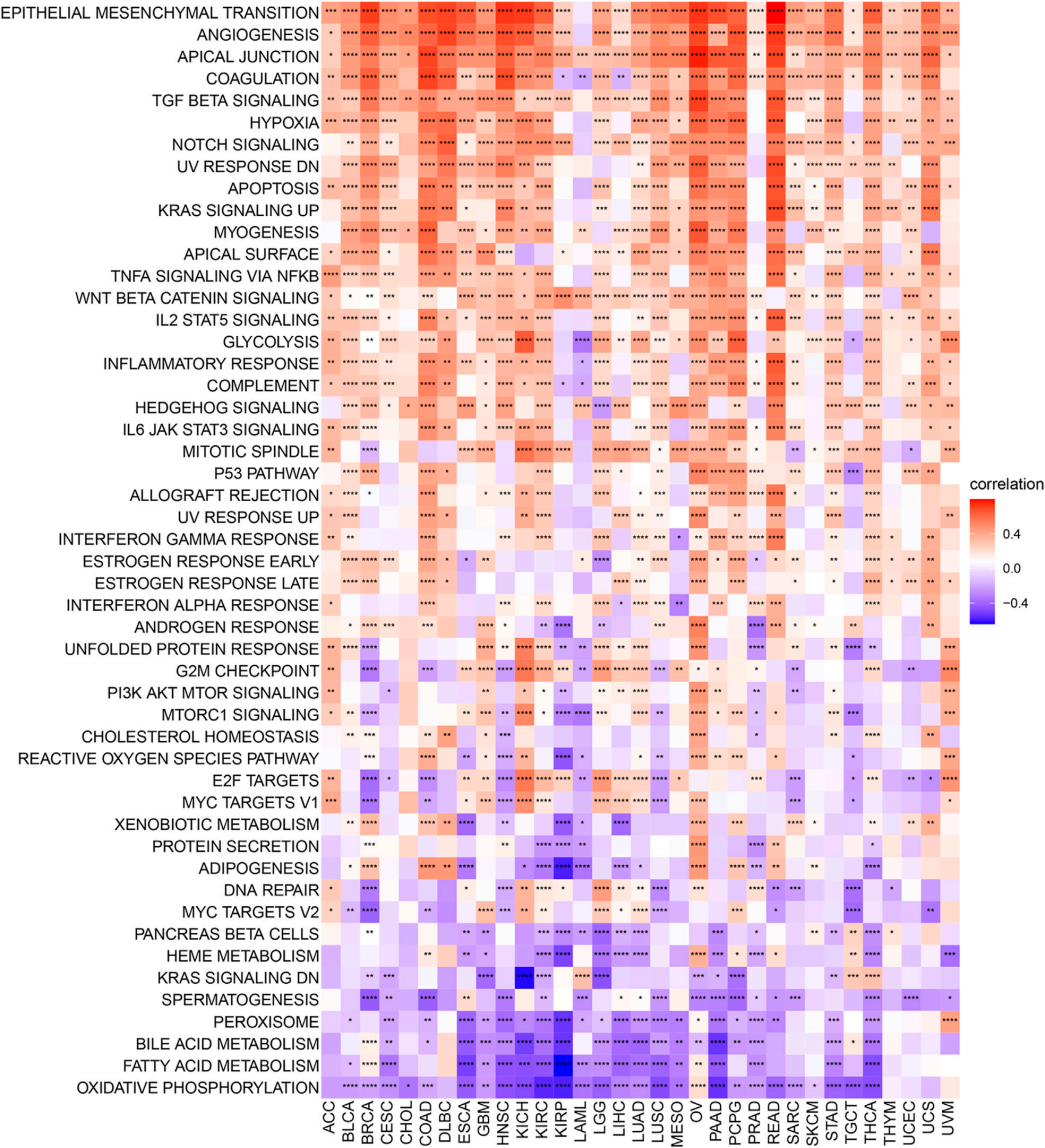
Heatmap for different hallmark pathway enrichment scores with C1QTNF6 expression level. *p < 0.05, **p < 0.01, ***p < 0.001, ****p < 0.0001.

The correlation between the expression level of *C1QTNF6* and the tumor pathway score was shown in Figure 4. We found that *C1QTNF6* is positively correlated with most cancer super-pathways. Among these pathways, oncogenic pathways such as angiogenesis and hypoxia showed a strong correlation with our *C1QTNF6* gene. A few pathways are negatively correlated with *C1QTNF6* expressions, such as Oxidative phosphorylation, fatty acid metabolism, and bile acid metabolism pathways.

### Correlation Analysis of *C1QTNF6* and Microenvironment Related Pathways

**FIGURE 5:**
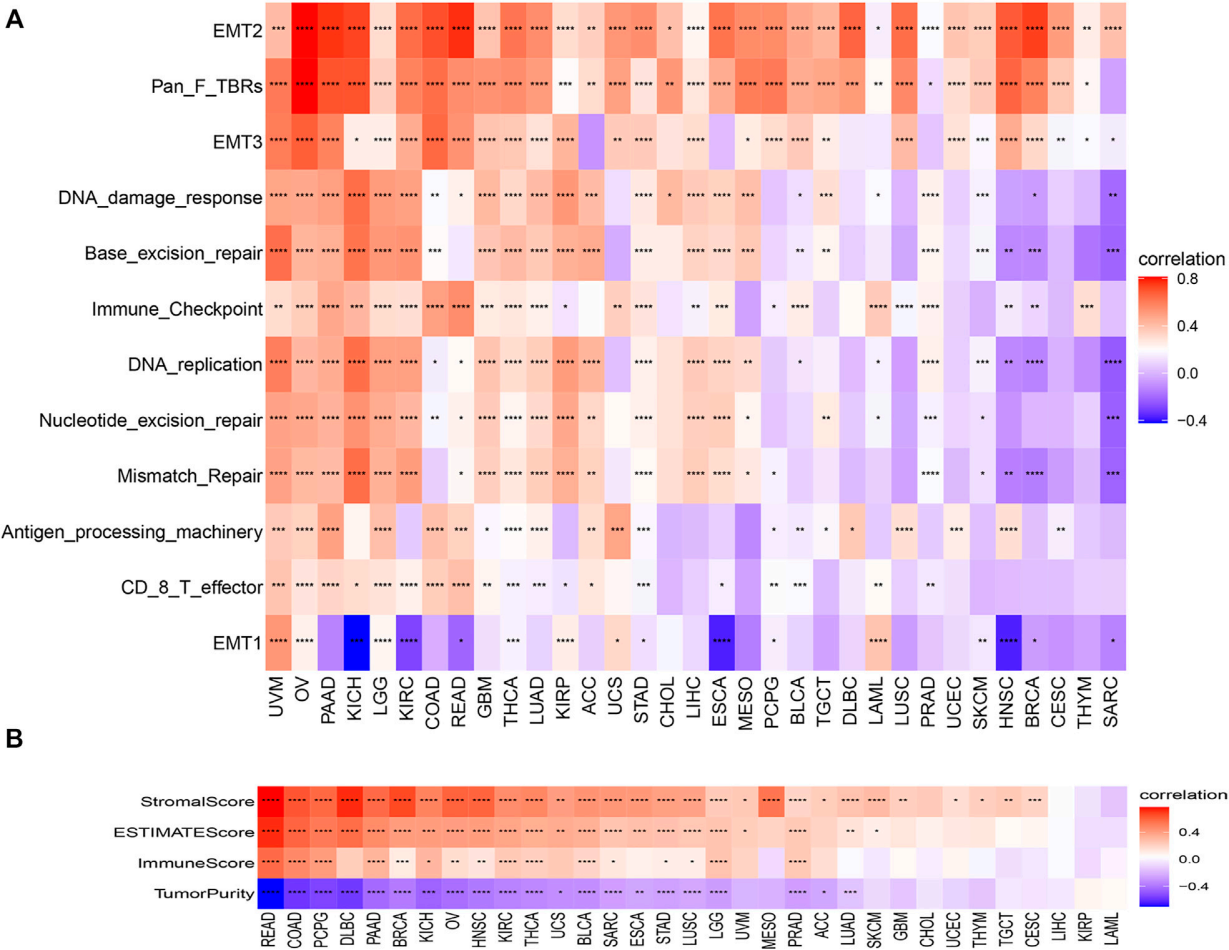
Tumor microenvironment role of C1QTNF6. **(A)** The correlations between the C1QTNF6 expression level and TMEp scores. **(B)** the correlations between the C1QTNF6 express level and ImmuneScore, StromalScore, and ESTIMATEscore. *p < 0.05, **p < 0.01, ***p < 0.001, ****p < 0.0001.

**FIGURE 6:**
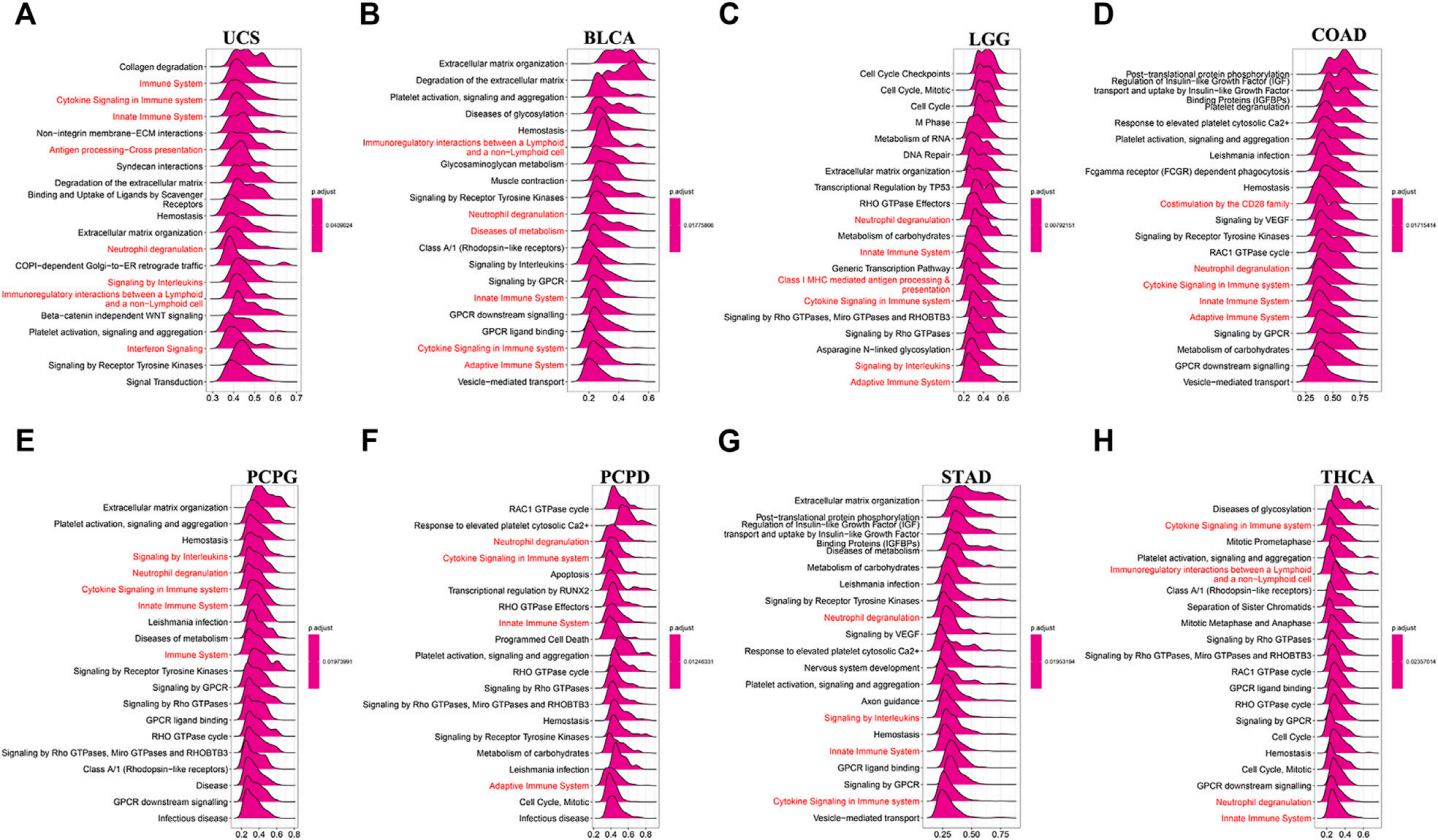
C1QTNF6’s GSEA results in TCGA pan-cancer. **(A–H)** The most 20 related pathways of GSEA are presented in the form of a mountain map, and immune-related pathways are marked in red.

We found that *C1QTNF6* was strongly correlated with TME related pathways, such as EMT1, base excision, and mismatch repair pathways correlated with the onset and progression of cancer (Figure 5A). Additionally, the activation of these pathways was highly correlated with the onset and progression of cancer, and this was highly consistent with our results in Figure 4. *C1QTNF6* gene expression was also significantly correlated with the immune checkpoint and CD8 T effector pathways (Figure 5A). The correlation between *C1QTNF6* and the immune score is shown in Figure 5B. StromalScore, Estimatescore, and immuneScore were all associated with *C1QTNF6*. *C1QTNF6* was found negatively correlated with These findings show that *C1QTNF6* could play a critical function in the control of biological activity in the immune milieu. Meanwhile, we explored the association between the infiltration of immune cells and *C1QTNF6* expression, and the findings were demonstrated in Supplementary Figure S2. We found the expression of *C1QTNF6* was negatively correlated with most immune cells, including B cell, Neutrophil, and CD8_T, and positively correlated with Monocyte and Macrophage. Next, GSEA was performed for the purpose of further examining the association of various pathways, especially immune-related pathways, with *C1QTNF6* in pan-cancer. The first 20 related pathways of GSEA are presented in the form of a mountain map, and immune-related pathways are marked in red (Figures 6A–H). For instance, in UCS, *C1QTNF6* was associated with the immune system, cytokine pathways, antigen processing-presentation, neutrophil degranulation, and interleukin immunomodulatory responses. These results further illustrate the important role of *C1QTNF6* in immune regulation.

### Correlation Between *C1QTNF6* and Immune-Related Genes

**FIGURE 7:**
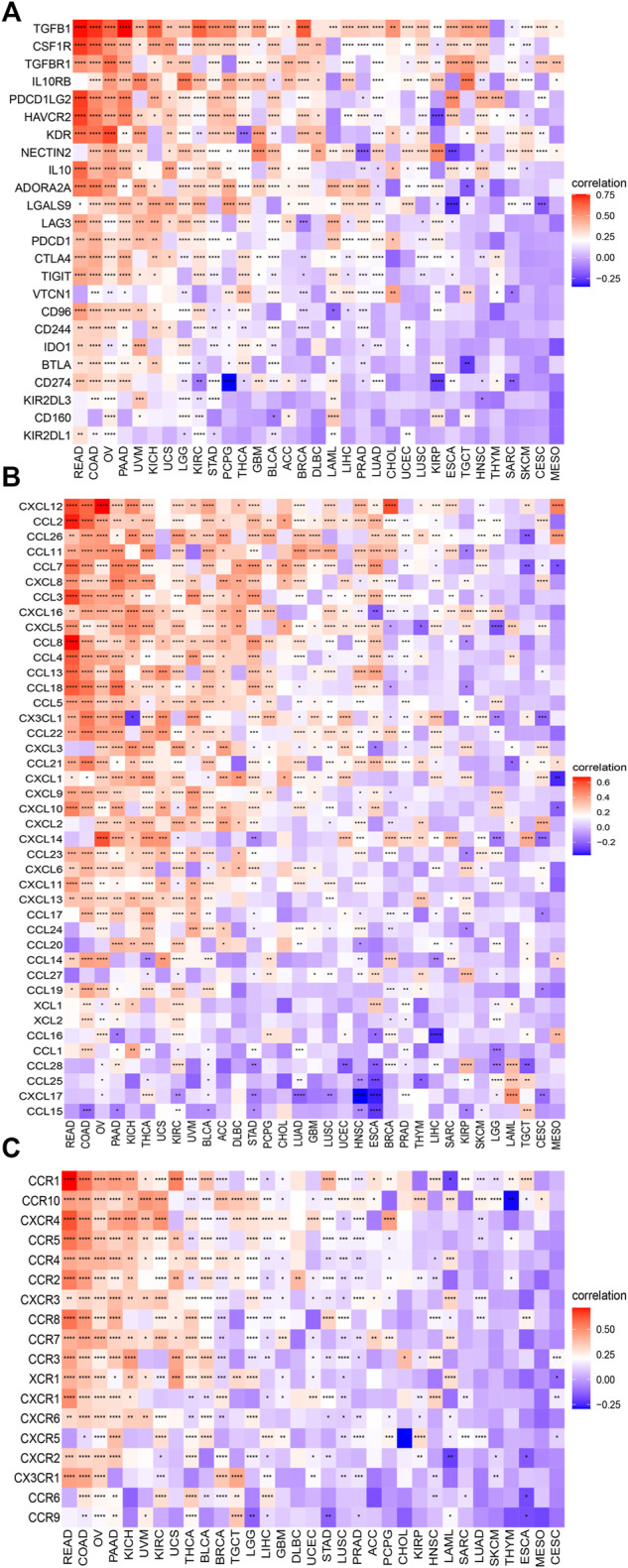
C1QTNF6 is correlated with immune-associated genes. The heatmap demonstrating the correlation between the C1QTNF6 expression level and **(A)** immunosuppressive genes. **(B)** chemokine genes. **(C)** chemokine receptors. *p < 0.05, **p < 0.01, ***p < 0.001, ****p < 0.0001.

We further explored the correlation between the *C1QTNF6* gene and genes associated with immune cells in pan-cancer. The results showed that *C1QTNF6* exhibited a positive association with most immunosuppressive genes, chemokines, and chemokine receptor genes (Figures 7A–C). Among the immunosuppressive genes, we found that the immune checkpoints including *LAG3*, *PDCD1*, *CTLA4*, and *TIGIT*, among the chemokines, there are *CXCL12*, *CCL2*, *CCL26*, and in chemokine receptors, we exhibited the *CCR1*, *CCR10*, *CXCR4*, etc.

### Drug Sensitivity Analysis

**FIGURE 8:**
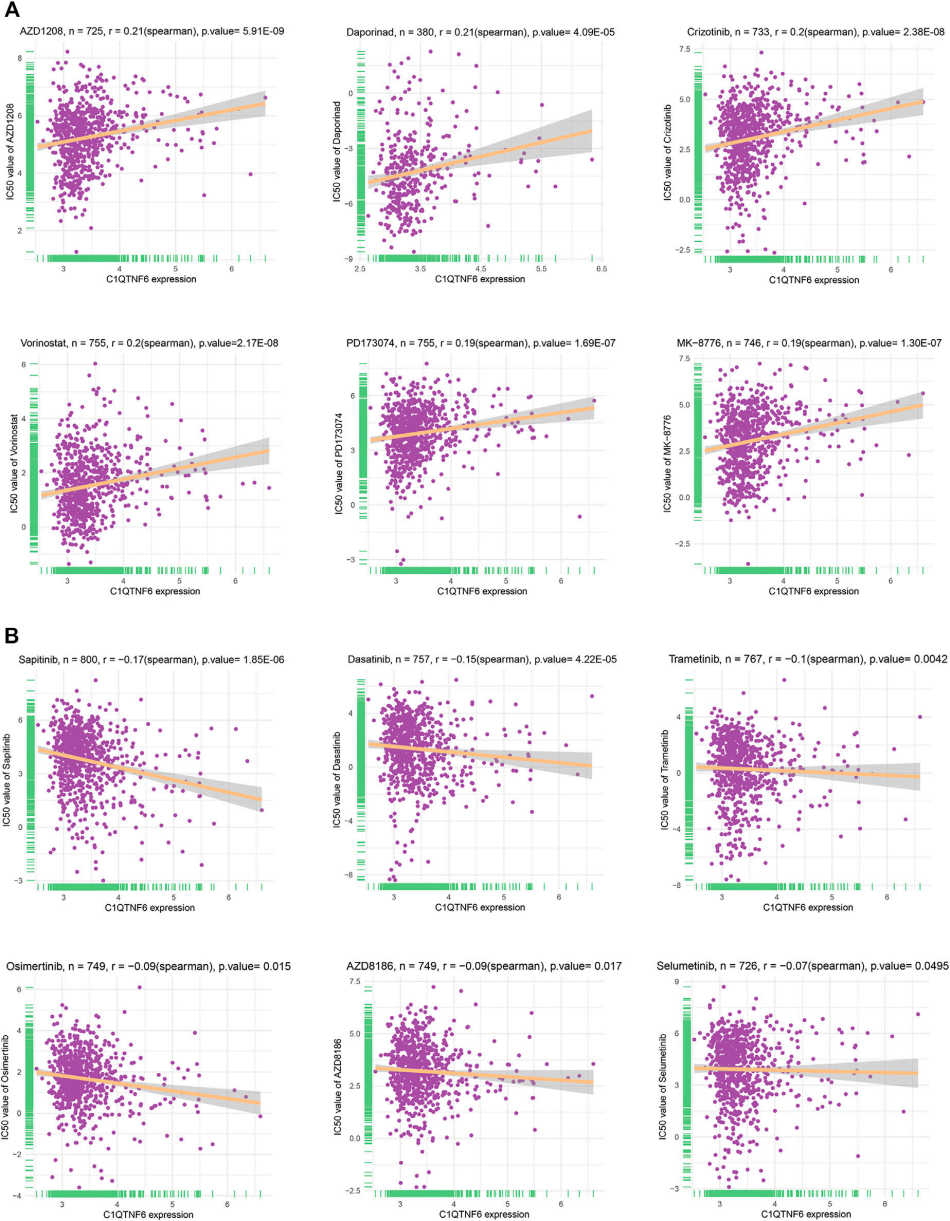
Drug sensitivity analysis in the form of correlation chart calculated by online tool GDSC2. **(A)** The top 6 positively correlated. **(B)** The only 6 negatively correlated.

A total of 198 drugs were identified as being associated with *C1QTNF6*. We showed the 6 drugs (Figure 8A) with the strongest positive correlation and the 6 drugs with the strongest negative correlation (only 6 negative, Figure 8B). The drugs that were identified to have positive correlation with *C1QTNF6* were namely AZD1208 (R = 0.21, *p* = 5.91E-09), Daprinad (R = 0.21, *p* = 4.09E-05), crizotinib (R = 0.2, *p* = 2.38E-08), Vorinostat (R = 0.2, *p* = 2.17E-08), PD173074 (R = 0.19, *p* = 1.69E-07), and MK-8776 (R = 0.19, *p* = 1.30E-07). In addition, six drugs were identified to be negatively correlated with C1QTNF6, namely Sapitinib (R = -0.17, *p* = 1.85E-06), Dasatinib e-05 (R = -0.15, *p* = 4.22), Trametinib (R = 0.1, *p* = 0.0042), Osimertinib (R = -0.09, *p* = 0.015), AZD8186 (R = -0.09, *p* = 0.017), and Selumetinib (R = -0.07, *p* = 0.0495). The results of the complete drug sensitivity analysis were presented in Supplementary Table S1. The commonly used anticancer drug in clinical treatment including Oxaliplatin, Cyclophosphamide, Cytarabine, Cisplatin, Cytarabine, Vinorelbine, Sorafenib, Docetaxel, and Fluorouracil were all less effective (higher IC50 value) in High expression of *C1QTNF6* groups (Supplementary Figure S3).

## Discussion

With the development of high-throughput bioinformatics, people’s understanding of genes or genomes has reached a new level. Exploration of molecular characterization of disease and individual genetic composition can facilitate clinical scientists to diagnose and treat the disease, which also facilitates the development of drug research and development, especially in cancer research. The discovery of a few genetic biomarkers has limited aid in the diagnosis and treatment of cancer, and identifying more biomarkers or combinations of biomarkers is increasingly important. In this study, we explore the role of *C1QTNF6* with comprehensive means using TCGA pan-cancer data.

Many studies have preliminarily elucidated the role of *C1QTNF6* in cancer. Qu et al. found that *C1QTNF6* is involved in promoting the proliferation and migration of gastric cancer cells and reducing apoptosis of gastric cancer cells (Qu et al., 2019). Han et al. found that *C1QTNF6* may be an independent prognostic factor for lung adenocarcinoma (Han et al., 2019). Song et al. discovered that *C1QTNF6* stimulates proliferation and attenuated apoptosis in oral squamous cell carcinoma (Song et al., 2021). Therefore, *C1QTNF6* might serve an instrumental function in the occurrence and progression of cancer. However, there has not been a comprehensive analysis of the significance of *C1QTNF6* in cancer.

We evaluated the *C1QTNF6* expression level in pan-cancer data and discovered that *C1QTNF6* is overexpressed in many types of cancer, such as LGG, LAML, LIHC, KIRP, LUAD, KIRC, LUSC, HNSC, PAAD, GBM, READ, ESCA, SARC, DLBC, SKCM, COAD, STAD, CHOL, THYM, BRCA, UCS, BLCA. Therefore, monitoring the expression level of *C1QTNF6* may be an effective diagnostic method for these cancers. Subsequently, the mutation analysis found that the alteration frequency of *C1QTNF6* in uterine carcinosarcoma was the highest. *C1QTNF6* had a strong positive correlation with CNA in CHOL, UCS, and PCPG. This enriched our understanding of the functionality of *C1QTNF6*. Survival analysis showed that *C1QTNF6* was an independent prognostic indicator for many tumors. Patient prognosis could be improved to a great extent as a result of this discovery. Analysis of the immune microenvironment revealed the role of *C1QTNF6* in the immune environment including immune-related gene and immune cells infiltration. The final drug sensitivity analysis provided the strongest association with *C1QTNF6*. This provides an idea for targeting *C1QTNF6* therapy.

Our study found that *C1QTNF6* is associated with a variety of classical pathways in many tumors, including epithelial-mesenchymal transformation (EMT), angiogenesis, apical junction, *TGF-β* signaling pathway, hypoxia, and Notch pathway. For instance, *C1QTNF6* had a significant correlation with EMT and angiogenesis. EMT has been widely recognized as a pathway through which cancer cells acquire malignant biological functions (Lamouille et al., 2014; Dongre and Weinberg, 2019). Through EMT, the potential of cancer cells to proliferate, invade and migrate is activated (Owusu-Akyaw et al., 2019; Pastushenko and Blanpain, 2019). In addition, EMT has been linked to drug resistance in many tumors (Du and Shim, 2016). Therefore, our study found a strong correlation between *C1QTNF6* and EMT, which could explain the poor prognosis after overexpression of *C1QTNF6* in many tumors. Angiogenesis which is one of the hallmarks of tumors is also necessary for tumor proliferation and invasion (Ramjiawan et al., 2017; Li et al., 2019). At present, antiangiogenic drugs have achieved preliminary results in the treatment of diverse solid tumors, but a considerable number of patients lack the persistent response of antiangiogenic drugs. Besides, our study found a significant positive correlation between *C1QTNF6* and angiogenesis, which is of reference value for us to further explore the mechanism of tumor angiogenesis and develop new therapeutic methods. In addition, TFN-ɑ SIGNALING VIA NFK-β and *IL2 STAT5* SIGNALING also showed a strong relationship with the *C1QTNF6* gene. The biological activity of *IL2* is related to T cell activation, CD8^+^ cytotoxicity, B cell activation, and the antitumor effects of enhanced macrophages, which all have been widely reported (Abbas et al., 2018).

Immune reprogramming is one of the hallmarks of cancer. Several therapies based on cancer immunity have been used clinically, such as immune checkpoints (Zhou et al., 2021). For instance, results of the bladder cancer clinical trial BLASST-1 showed that Nivolumab (Opdivo) in combination with gemcitabine and cisplatin neoadjuvant therapy in patients with muscle-infiltrating bladder cancer (MIBC) achieved a significant effect: a pathology complete response rate (pCR) of 49% (Gupta et al., 2020). Therefore, identifying the changes in the cancer-immune microenvironment not only helps us understand the pathogenesis of cancer but also promotes the development of cancer immunotherapy (Huang et al., 2018). According to the findings of the present research, *C1QTNF6* serves a prominent function in the immune milieu of cancer cells. It is significantly linked to diverse immune pathways and immune components. In the TME, stromal and immune cells are the two most crucial kinds of non-tumor constituents, and both have been considered to play a crucial role in the diagnosis and prognostic evaluation of cancers. In tumors, immune and stromal scores can be used to aid in the quantification of immunological and stromal components that are present (Bruni et al., 2020). *C1QTNF6* shows a great correlation with the ESTIMATEscore, StromalScore, and ImmuneScore. Besides, *C1QTNF6* is obviously correlated with many immune checkpoints including *LAG3*, *PDCD1*, *CTLA4*, which suggested that *C1QTNF6* may act as a new immune checkpoint for tumor immunity. Meanwhile, our results show that *C1QTNF6* also have markable relation with chemokines and chemokines receptor such as *CCL22*, *CCL5*, etc. *CCL22* secreted by the M2 macrophages may recruit Treg T cells, which suppressed the immune response (Atri et al., 2018). *CCL5* is a key chemokine for CD8^+^ T cells to enter tumor cells (Dangaj et al., 2019). *C1QTNF6* shows a significant association with the infiltration of monocyte, Macrophage, B cells, Monitoring the *C1QTNF6* expression may reflect the degree of infiltration of these immune cells, it is noteworthy that *C1QTNF6* is not manifest a strong correlation to all the immune cells because the activation, chemotaxis, and infiltration of immune cells are regulated by gene networks, especially in pan-cancer analysis. The above results indicate that *C1QTNF6* has significance for further exploration in immune regulation in the tumor microenvironment. These findings were further validated by the GSEA and correlation exploration between *C1QTNF6* and immune cells. In the drug sensitivity analysis, we found higher *C1QTNF6* expression in tumors indicates a higher IC50 value for most drugs in the GDSC2 database, which shows the measurement of *C1QTNF6* expression level may act as a reliable indicator for clinical therapy. P-gp, a well-known multidrug resistance protein, encoded by MDR1, it serve as medicine pump by reverse the concentration of lipophilic drugs with positive charge inside the cell to the outside of the cell, so that the intracellular chemotherapy drugs can not reach the effective concentration and develop drug resistance (Robey et al., 2018). In non-small-cell-lung cancer, MET amplification is thought to be responsible for resistance to targeted drugs in EGFR-mutation-positive patients, which results in malignant biological behavior of tumors, such as invasion, metastasis, escape from apoptosis (Coleman et al., 2021). Combining with the *C1QTNF6* expression and its drug sensitivity analysis (positively correlated and negatively correlated), we may establish risk stratification for cancer patients, which optimize the development and application of anti-cancer drugs. These findings suggest that *C1QTNF6* may be a potential target for cancer therapy, which make contribution to study the mechanisms of anti-cancer drugs resistance.

Cancer immunotherapy has recently been in full swing. Effective identification of potential tumor antigens has contributed to the development of immunotherapy. These tumor antigens are often associated with more copy number mutations or methylation changes. Our study provides the mutation landscape of *C1QTNF6* in pan-cancer and the correlation landscape with copy number variation and methylation. Furthermore, we identified a correlation between *C1QTNF6* and chemokine receptors. All these results provide a reference that *C1QTNF6* can be used not only as an indicator of the TME, but also as a highly effective indicator of immune indicators.

Overall, our study is the first pan-cancer analysis of *C1QTNF6*. Compared with other studies, our study provided a landscape of the prognosis and immunity correlation of *C1QTNF6* in a variety of cancers, which is conducive to the precise treatment of cancer. However, there are also limitations in our study. We lack experiments to verify our conclusions, which we will improve in the future.

## Conclusion

Overall, we identified the value of *C1QTNF6* in pan-cancer by multiple analyses. *C1QTNF6*, as a promising prognostic biomarker, manifests its promising prospect in immunoregulation and a potential target for tumor therapy.

## Data Availability

Publicly available datasets were analyzed in this study. This data can be found here: This study used online resources, which are available from the TCGA database, GTEx through USUC Xena at https://xena.ucsc.edu, the cBioportal database at http://www.cbioportal.org/, and GDSC2 at https://www.cancerrxgene.org/.

## Supplementary Material

Supplementary Figure S1 Univariate cox regression and multivariate cox regression analysis of C1QTNF6 and other clinical features in KIRC, LUAD, LGG, KIRP, ACC, UVM, MESO, LIHC, HNSC, KICH, BLCA, THCA, UCEC.

Supplementary Figure S2 Correlation between the expression of C1QTNF6 and the infiltrating levels of immune cell. **p* < 0.05, ***p* < 0.01, ****p* < 0.001, *****p* < 0.0001.

Supplementary Figure S3 Drug sensitivity of commonly used anti-cancer drugs in clinical use in the forms of boxplot charts calculated by online tool GDSC2. **(A–I)** Oxaliplatin, Cyclophosphamide, Cytarabine, Cisplatin, Cytarabine, Vinorelbine, Sorafenib, Docetaxel, Fluorouracil.

Supplementary Table S1 Drug sensitivity differences according to the C1QTNF6 expression of all the 198 drugs in the GDSC2 database.
