# Supplementary figures and images for: C1QTNF6 is a Prognostic Biomarker and Related to Immune Infiltration and Drug Sensitivity: A Pan-Cancer Analysis
Source: Front Pharmacol. 2022 Mar 23;13:855485. doi: 10.3389/fphar.2022.855485 (PMC8985594; doi:10.3389/fphar.2022.855485)

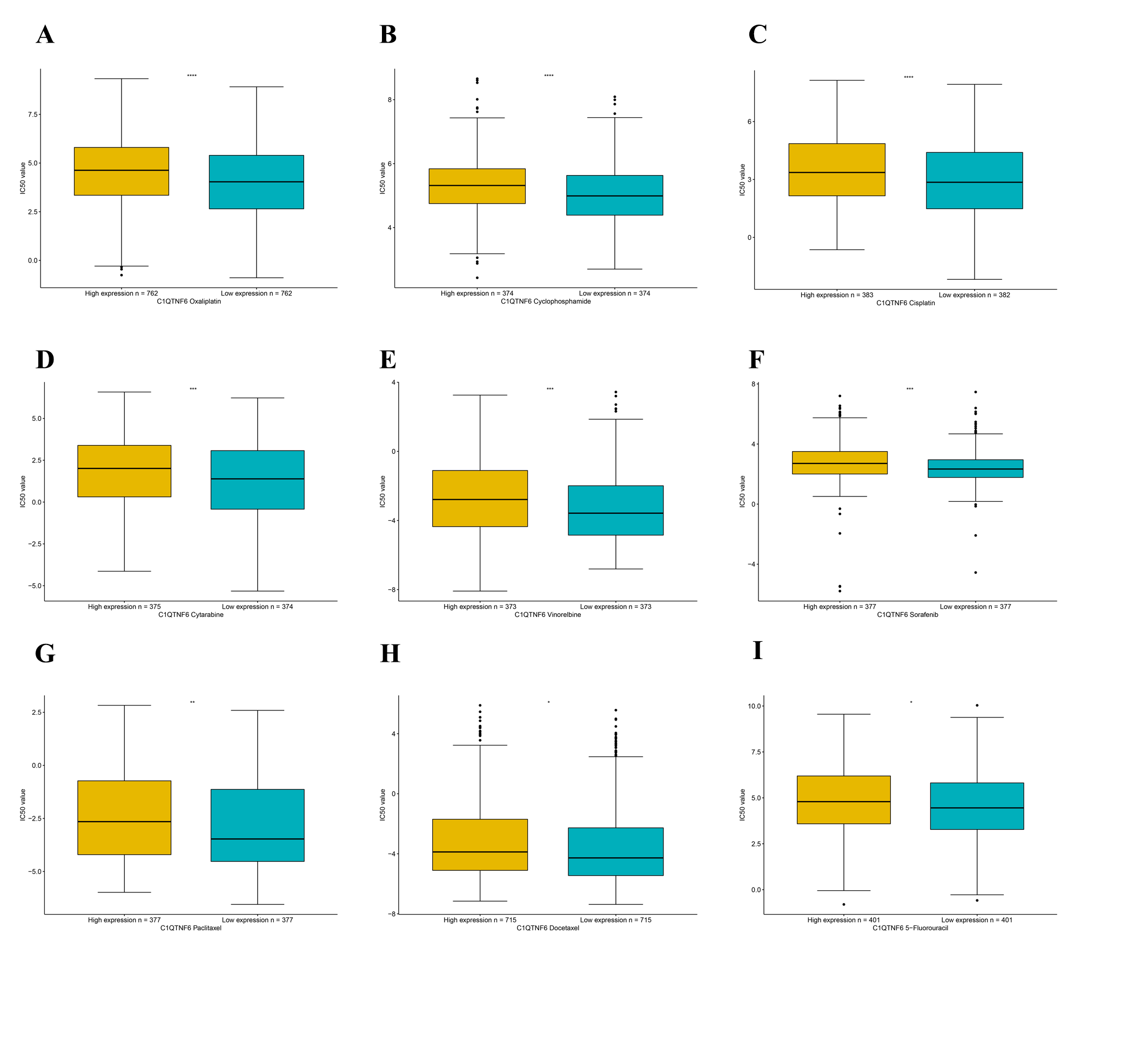

Supplement: Supplementary file 1 [file Image3.TIF]

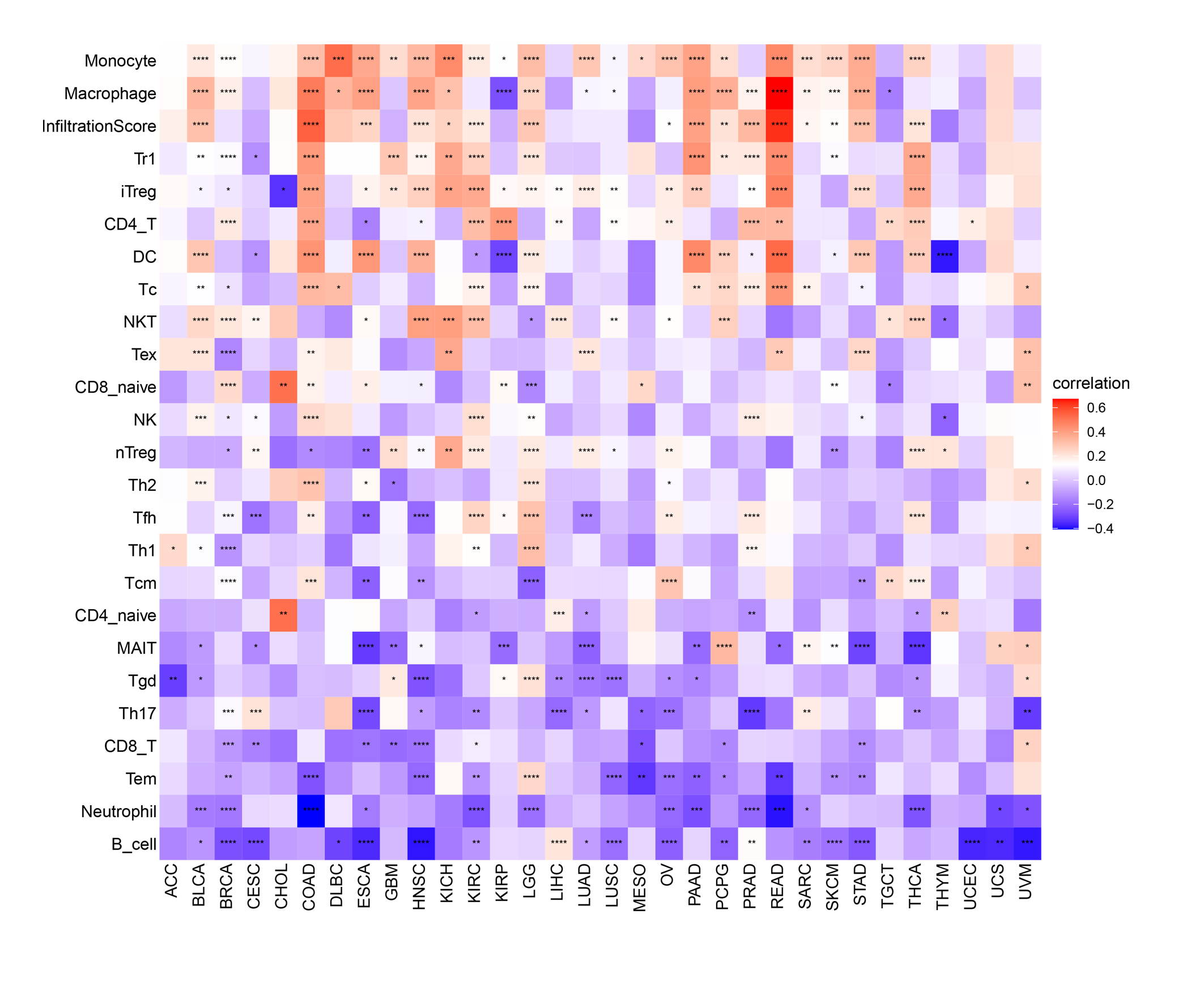

Supplement: Supplementary file 2 [file Image2.TIF]

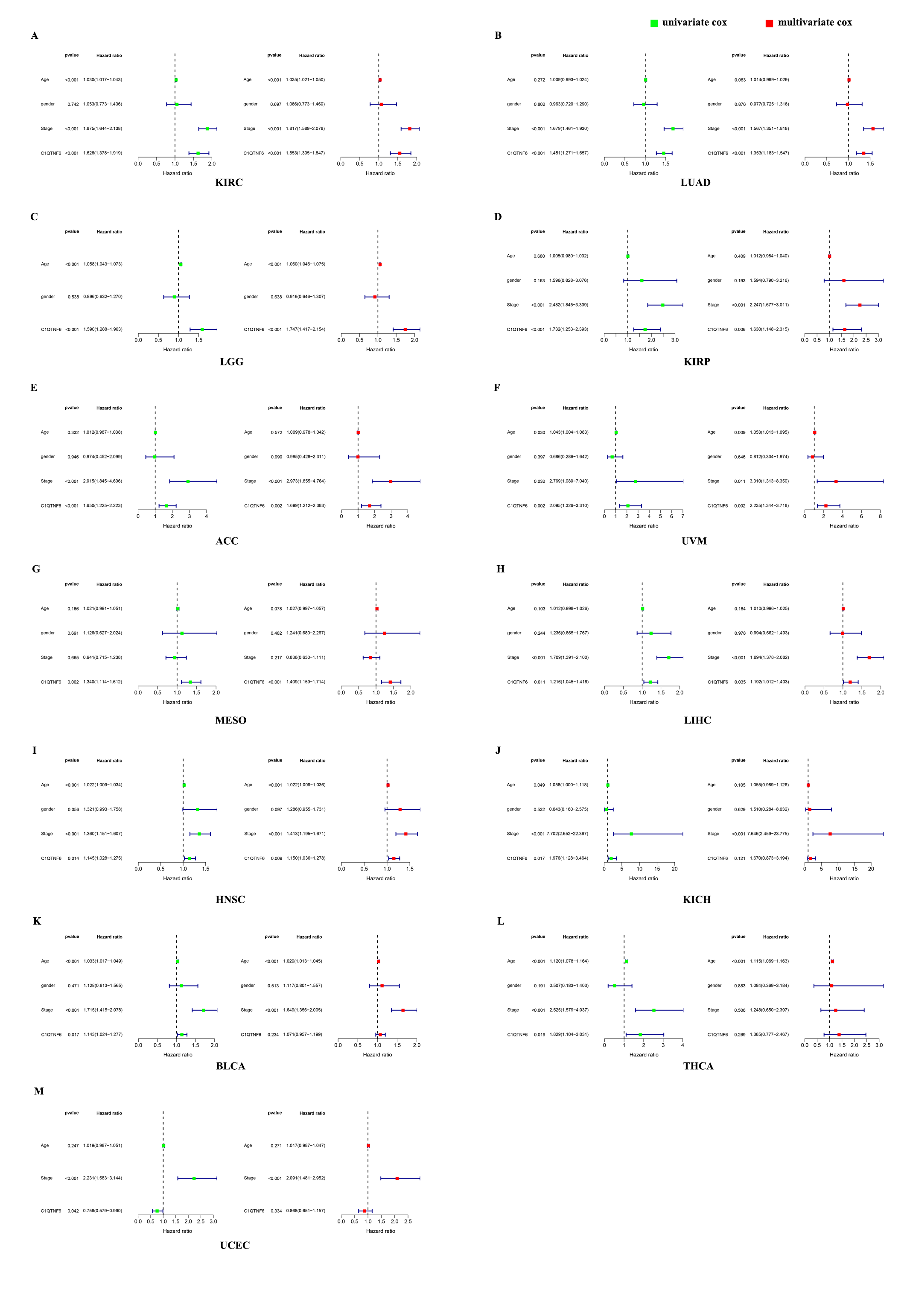

Supplement: Supplementary file 3 [file Image1.TIF]
